# Bayesan Model to Predict R Status After Neoadjuvant Therapy in Pancreatic Cancer

**DOI:** 10.3390/cancers16234106

**Published:** 2024-12-07

**Authors:** Isabella Frigerio, Quoc Riccardo Bao, Elisa Bannone, Alessandro Giardino, Gaya Spolverato, Giulia Lorenzoni, Filippo Scopelliti, Roberto Girelli, Guido Martignoni, Paolo Regi, Danila Azzolina, Dario Gregori, Giovanni Butturini

**Affiliations:** 1 Hepato-Biliary and Pancreatic Surgery Unit, Pederzoli Hospital, 37109 Peschiera del Garda, Italy; isabella.frigerio@ospedalepederzoli.it (I.F.); elisa.bannone@ospedalepederzoli.it (E.B.); alessandro.giardino@ospedalepederzoli.it (A.G.); filippo.scopelliti@ospedalepederzoli.it (F.S.); roberto.girelli@ospedalepederzoli.it (R.G.); paolo.regi@ospedalepederzoli.it (P.R.); giovanni.butturini@ospedalepederzoli.it (G.B.); 2 Collegium Medicum, University of Social Sciences, 90-136 Łodz, Poland; 3 General Surgery 3, Department of Surgical Oncological and Gastroenterological Sciences, University of Padova, 35128 Padova, Italy; quocriccardo.bao@unipd.it; 4 Unit of Biostatistics, Epidemiology and Public Health, Department of Cardiac, Thoracic, Vascular Sciences and Public Health, University of Padova, 35128 Padova, Italy; giulia.lorenzoni@ubep.unipd.it (G.L.); danila.azzolina@ubep.unipd.it (D.A.); dario.gregori@ubep.unipd.it (D.G.); 5 Department of Pathology, Pederzoli Hospital, 37109 Peschiera del Garda, Italy; guido.martignoni@ospedalepederzoli.it

**Keywords:** R Status, Bayesan model, predictive model, pancreatic cancer, neoadjuvant therapy

## Abstract

Objective: To build a Bayesian approach-based model to predict the success of surgical exploration post-neoadjuvant treatment. Background: Pancreatic cancer (PDAC) is best treated with radical surgery and chemotherapy, offering the greatest chance of survival. Surgery after neoadjuvant treatment (NAT) is indicated in the absence of progression, knowing the limits in accurately predicting resectability with traditional radiology. R Status being a pathological parameter, it can be assessed only after surgery. Method: Patients successfully resected for histologically confirmed PDAC after NAT for BR and LA disease were included, with attention to the predictors of R status from the existing literature. The Bayesian logistic regression model was estimated for predicting the R1 status. The area under curve (AUC) of the average posterior probability of R1 was calculated and results were reported considering the 95% posterior credible intervals for the odds ratios, along with the probability of direction. Results: The final model demonstrated a commendable AUC value of 0.72, indicating good performance. The likelihood of positive margins was associated with older age, higher ASA score, the presence of venous and/or arterial involvement at preoperative radiology, tumor location within the pancreatic body, a lack of tumor size reduction post-NAT, and the persistence of an elevated Ca19.9 value. Conclusions: A Bayesian approach using only preoperative items is firstly used with good performance to predict R Status in pancreatic cancer patients who underwent resection after neoadjuvant therapy.

## Simple Summary

A Bayesian model was developed to predict surgical success after neoadjuvant treatment for pancreatic cancer. The model showed good performance (AUC 0.72) and offers an innovative and reliable method to predict the likelihood of negative margin in PDAC patients after neoadjuvant chemotherapy.

## 1. Introduction

Pancreatic ductal adenocarcinoma (PDAC) is a highly lethal malignancy [1], with radical surgery combined with chemotherapy and eventually chemoradiotherapy offering the best chance for prolonged survival [2]. Neoadjuvant treatment (NAT) is recommended for borderline resectable (BR) and locally advanced (LA) PDAC [3,4]. Even in initially resectable cases, particularly those with high-risk features, NAT is increasingly considered to obtain radical resections [5]. Achieving microscopic clearance of surgical margins (R0 resection) remains the primary surgical goal, as it is independently associated with improved survival [6,7,8].

While NAT has been shown to increase both resection rates and the likelihood of R0 resection [9], the inherent limitations of radiological imaging in accurately predicting treatment response and resectability create challenges [10,11]. Surgical exploration is often necessary when there are no clear signs of disease progression [12]. This raises the imperative for alternative predictive models in the preoperative setting to identify patients suitable for surgical exploration or those who may benefit from extended NAT or a change in chemotherapy regimen.

The Bayesian approach allows for the integration of existing knowledge on outcomes in PDAC resection after NAT and incorporates assumptions about the probability distributions of factors influencing margin status. This approach permits more nuanced predictions regarding the risk of achieving an R1 resection based on individual patient characteristics. The adoption of the Bayesian approach represents a scientific effort to improve our ability to predict the success of surgical exploration after NAT in PDAC patients, thereby supporting more informed clinical decision-making in this challenging domain of pancreatic cancer management.

This study aims to develop a preoperative model to predict the risk of failure of surgical exploration following NAT, where failure is defined as achieving an R1 resection.

## 2. Methods

The study protocol was notified and approved by the local ethical committee (Comitato Etico per la Sperimentazione Clinica delle Province di Verona e Rovigo CESC 4226).

### 2.1. Inclusion and Exclusion Criteria

All patients who underwent surgical resection after NAT between 2010 and 2020 at a high-volume pancreatic surgery unit were retrieved from a prospectively maintained institutional database. Informed consent was obtained in accordance with the ethical standards of the committee on human experimentation of the institution in which the experiments were performed or in accord with the ethical standards of the Helsinki Declaration of 1975.

The study included patients who underwent resection for histologically confirmed PDAC after NAT for BR and LA disease at diagnosis. Given the study period and the guidelines in place at that time, according to institutional policy, no resectable PDAC was treated with NAT and therefore was not included in this study.

Patients with the following characteristics were excluded: upfront resection; surgery for other malignancy (i.e., ampullary, periampullary, duodenal, neuroendocrine tumor), or benign disease.

### 2.2. Data Collection

Data from our database were extracted, focusing on preoperative items. We included demographic and clinical characteristics (including CA19-9 levels, DCA19-9 (defined as the difference between the CA19-9 baseline and CA19-9 at restaging), NAT regimen, and radiological features (including baseline stage according to NCCN guidelines, tumor size, and vascular involvement). Comorbid conditions were collected and summarized using the Charlson Comorbidity Index (CCI) [13]. Surgical details including the type of resection, vascular resection, estimated blood loss and postoperative data including length of stay and postoperative complications (e.g., postoperative pancreatic fistula, post-pancreatectomy hemorrhage, and delayed gastric emptying according to ISPGS definitions) [14,15,16] were also collected. The severity of complications was classified according to the Clavien-Dindo score [17].

All histopathologic specimens were reviewed by a dedicated pancreatic pathologist to confirm TNM staging and margin (R) status. The pathologic stage was classified according to the AJCC 8th Edition [18], and an R1 margin was defined as the presence of invasive carcinoma within 1 mm of any of the surgical specimen margins [19].

### 2.3. Statistical Analysis

The descriptive statistics were reported according to the R0 margin by summarizing the continuous data as a median and interquartile range (IQR); categorical data are instead reported as absolute frequencies and percentages. Wilcoxon-type tests were performed for continuous variables, and the Pearson chi-square test, or Fisher’s exact test when appropriate, for the categorical variables.

The Bayesian logistic regression model was estimated for predicting the R1 status. The covariates included in the models were extracted based on the literature and clinical knowledge: (1) age at intervention; (2) American Society of Anesthesiologists (ASA) physical status classification system score; (3) arterial involvement; (4) post-NAT Tumor diameter; (5) venous contact; (6) tumor location in the pancreas; (7) preoperative radiotherapy; and (8) preoperative Ca 19-9 levels. Different scenarios were considered for model development by setting the priors as uninformative or informative.

Weakly informative. The prior parameters on the log odds coefficients *β*! were defined as *β*! ∼ normal (*μ* = 0, *σ* = 2.5/*s*”) where *s*” is the standard deviation of the predictor. This approach is suggested in the literature to adjust the scales (standard deviation) of the priors based on the data and to maintain a vague prior definition also accounting for the nature of the data [20].

Informative priors based on the literature. The informative priors were derived by eliciting the 0.025, 0.5, and 0.975 quantiles by using the 95% log odds confidence intervals for the R1 predictors identified in the literature. The elicitation process was performed by considering the elicited probabilities as inputs and fitting normal distributions using least squares on the cumulative distribution function via SHELF [21] approach.

Within the informative priors scenarios, a power prior [22] approach was also considered to discount the prior information on the final inference. The prior scale parameters were defined for each predictor by using a discounting factor of *δ* as *β*! ∼ normal (*μ* = 0, *σ*(1 + *δ*)). For example, a *δ* value of 0.5 increased the prior variance by 50%, also reducing the prior weight on the final inference.

The inference for typical discounting factors of 50% and 90% were reported together with the model results for the discounting factors stabilizing the model’s predictive performance according to the *δ* values. The *δ* equilibrium point was selected in correspondence with the inflection point of the calculated AUC according to *δ* values ranging from 0.1 to 0.95.

Predictive performance. The average posterior predictive distribution for the R1 probability was computed for each subject. The area under curve (AUC) of the average posterior probability of R1 was calculated. The predictive AUC accounting for the prior discounting factors ranging from 0.10 to 0.9 was calculated. The Bayesian model results were reported for the model considering the discounting factor and maximizing the predictive accuracy.

The models were tuned in 1000 iterations in four chains; the convergence was visually inspected on the trace plots. The analyses were carried out in R with rms [23] and brms [24] packages.

Model results. The results were reported considering the 95% posterior credible intervals for the odds ratios, along with the probability of direction (p_d), also known as the Maximum Probability of Effect (MPE). This varies between 50% and 100% (i.e., 0.5 and 1), and can be interpreted as the probability (expressed as a percentage) that a log odds *β*! parameter is strictly positive or negative. Mathematically, it is defined as the proportion of the posterior distribution that is the same sign as the median. Although expressed differently, this index is quite similar to the frequentist *p*-value.

## 3. Results

### 3.1. Patients’ Characteristics

Preoperative details are summarized in Table 1. Overall, 205 patients with PDAC who underwent surgical resection after NAT at the Pancreatic Surgical Unit at Pederzoli Hospital were included. Of these, 100 (49%) were female. The median age at the surgery of the cohort was 64 (IQR 56–70) years. At baseline, 64% were staged as BR, and 36% as LA. The majority underwent NAT with FOLFIRINOX (n = 81, 46%), or Gemcitabine + n-ab-paclitaxel (n = 55, 31%), and 51 patients (25%) also received radiotherapy. After NAT, 149 patients (72.7%) underwent pancreaticoduodenectomy, 49 (23.9%) distal pancreatectomy, and 7 (3.4%) total pancreatectomy. In all patients, intraoperative frozen section analysis of the pancreatic stump was performed, and total pancreatectomies were carried out only after multiple positive results from the frozen section analysis. Of the resected patients, 150 (73.2%) achieved an R0 resection, while 55 (26.8%) had an R1 resection. Vascular resection was required in 49 patients (24%). Major postoperative complications occurred in 28 patients (14%), with 18 (9%) requiring reoperation, and postoperative mortality occurred in 2 patients (1%).

### 3.2. Pathological Details

As summarized in Table 2, R0 resection was achieved in 150 patients (73.3%). Among patients with R1 resection, 39 (71%) had retroperitoneal margin involvement. R1 patients also had larger tumors confirmed by pathology (25 mm vs. 20 mm, *p* = 0.001), and higher locoregional nodal involvement (74% vs. 48%, *p*: 0.003). R0 resections were associated with higher rates of major and complete pathological response (33% and 11% vs. 7% and 0%, *p* < 0.001).

### 3.3. Comparison Between R0 and R1 Resection

When comparing R0 and R1 groups, R1 patients were older (66 (IQR 61–70) vs. 62 (IQR 54–70), *p* = 0.02), and had a higher CCI (CCI > 4, 39 (89%) vs. 90(68%), *p* = 0.007). Jaundice at diagnosis was more common in R1 than R0 patients (58 vs. 41%, *p* = 0.014).

While no difference was documented at baseline between the two groups, after NAT, R1 patients had larger radiological median tumor sizes (30 (IQR 23–35) vs. 24 (IQR 18–29) mm, *p* < 0.001), and more frequent vascular involvement (73 vs. 51%, *p* = 0.006). They also showed lower DCA19-9% (63% vs. 86%, *p* = 0.020), and fewer CA19-9 responders (16% vs. 64%, *p* = 0.004).

No significant differences were observed in the tumor site, NAT type, or radiotherapy administration. R1 resections were associated with longer surgery (390 min vs. 360 min, *p* = 0.030), more frequent vascular resections (38% vs. 19%, *p* = 0.004), greater intraoperative blood loss (450 vs. 300 mL, *p* = 0.006), and higher transfusion rates (33% vs. 9.4%, *p* > 0.001). R1 patients experienced a higher incidence of major postoperative complication (27% vs. 8.7%, *p* > 0.001), including Grade 3 PPH (13% vs. 2.9%, *p* = 0.028), sepsis (9.4% vs. 1.3, *p* = 0.014) and reintervention (17% vs. 6.1%, *p* = 0.027). Postoperative mortality was 1%, with no differences between the groups.

### 3.4. Overall Survival

**Figure 1:**
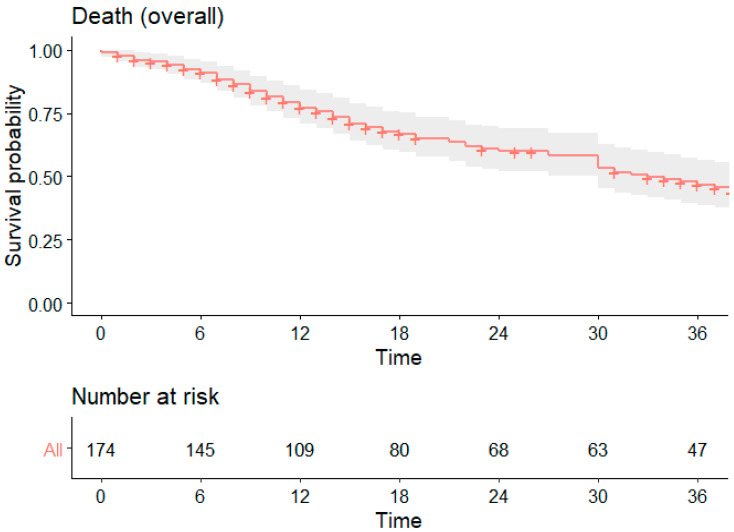
Overall survival.

**Figure 2:**
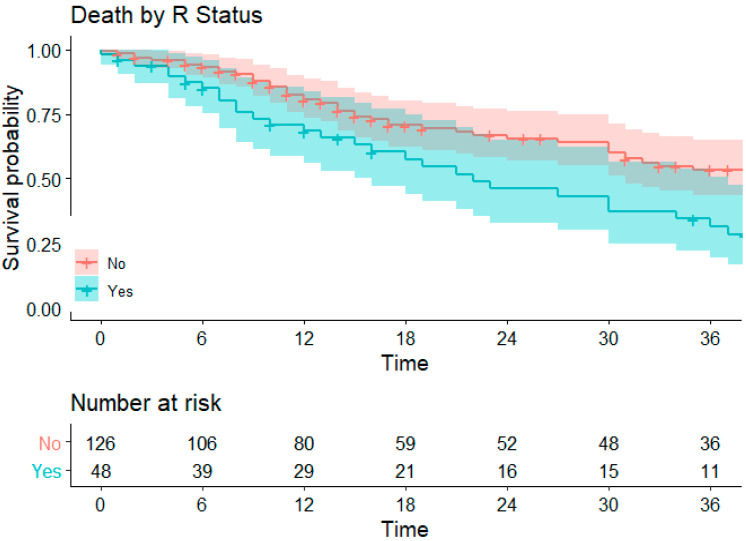
Survival by R status.

The estimated 1-, 2-, and 3-years OS for the entire cohort was 77%, 50%, and 46%, respectively (Figure 1). When comparing R0 and R1 groups, the 1-, 2-, and 3-years OS was 80%, 65%, and 53% for R0, and 68%, 46%, and 31% for R1 (*p* = 0.008; HR 1.79 [1.16–2.77]) (Figure 2).

### 3.5. Results of Bayesian Model Development

The final model showed good performance in predicting the risk of R1 resection with an AUC of 0.72 (Supplementary Material S1). Factors significantly associated with the likelihood of an R1 resection are shown in Table 3 and included older age, higher ASA score, the presence of venous and/or arterial involvement on preoperative imaging, tumor localization in the pancreatic body, a lack of tumor size reduction post-NAT, and the persistence of elevated Ca19.9 levels. Detailed results for various prior scenarios (Supplemental Material S1), confirmed the consistency of the final model’s findings.

## 4. Discussion

This study introduces a novel Bayesian approach for predicting R status in pancreatic cancer patients who underwent preoperative therapy. The use of Bayesian modeling is substantiated by an extensive body of literature that identifies potential predictors of R status in pancreatic surgery and highlights its prognostic implications. By leveraging existing knowledge, the Bayesian approach enhances the precision of predicting the R status in patients with PDAC undergoing resection following preoperative therapy [25,26,27].

Conventional predictive models often rely solely on the author’s data and clinical expertise. In contrast, the Bayesian approach can offer significant advantages by integrating pre-existing evidence into model development. This systematic framework provides a valuable opportunity for new predicting models by combining current knowledge with accumulated evidence, potentially leading to more accurate and relevant predictive outcomes.

The focus on R status aligns with its well-established role as an independent prognostic factor for survival [28,29,30]. Despite earnest endeavors to improve preoperative staging, its reliability remains inherently limited due to its dependance on macroscopic findings alone. As a result, positive microscopic margin after surgical resection persists in a substantial proportion of cases—up to 80% of upfront surgeries and around 40% of resections following NAT [31,32]. Within this series, 73% of patients achieved an R0 resection, which is consistent with rates reported following NAT.

This analysis focused on a specific cohort of PDAC patients who showed no progression following NAT, with positive response indicators, such as reduced Ca19.9 levels, tumor size, and radiological stage. The relationship between NAT and R status remains a topic of debate in the literature. While some studies have demonstrated an association between R status and OS independently of NAT, others have reported improved outcomes in patients who underwent NAT compared to those who did not, even when stratified by the same R status [7]. In our series, R0 patients exhibited superior surgical, pathological, and oncological outcomes, likely attributed to less advanced disease and/or a better response to chemotherapy. While the impact of R status on OS remains debated with varying consensus in the literature [33,34], recent evidence including findings from this study strongly supports its impact on outcomes, underscoring the need for improved preoperative assessment methods.

Previous research has explored predictive models. Bolm et al.’s identification of baseline radiological perivascular superior mesenteric artery stranding as a predictor of R status underscores the potential of imaging factors in shaping prognostic insights [10]. Notably, our study aligns with these findings, demonstrating a 33% likelihood of margin-positive (R1) resection and a subsequent unfavorable prognosis in the presence of SMA straining. Similarly, Toesca et al. [35] proposed a preoperative resectability scoring system, particularly applicable to patients undergoing upfront surgery, based on the radiological extension of vascular involvement which strongly correlated with achieving R0 resection. Maulat et al.’s preoperative resectability score [36] excluded patients receiving NAT and included a broad spectrum of preoperative factors besides radiological findings, such as tumor size, but overlooked parameters like CA19-9 value, thus potentially limiting its real-world applicability. In our study, neither tumor size nor radiological staging at first diagnosis were associated with R status or OS, in contrast to their prognostic value when considered after NAT. This disparity underscores the need for a predictive model that overcomes the limitations of conventional radiological assessments, emphasizing the pursuit of more reliable and nuanced prediction methods.

In the future, radiomics will likely fill the gap and improve preoperative assessment for PDAC patients after NAT [37,38], but it remains expensive and still under investigation.

As emphasized by Strijker [39], there is an urgent need for more refined predictive models to guide the management of pancreatic cancer patients and avoid futile or high-risk resection.

Oba et al. [40] built a nomogram, using the National Cancer Database to predict survival based on preoperative baseline factors, shifting for the first time the concept of resectability from anatomical and radiological features to survival-based predictions.

Our study takes a step further by introducing a novel approach for predicting R status in PDAC patients undergoing surgery after NAT, integrating the existing literature and clinical knowledge. The use of Bayesian modeling highlights the scientific novelty of these results and allowed for the identification of the most significant preoperative variables, as recognized in the literature. Unlike traditional models that capture a single snapshot in the disease course, this approach aims to provide a more comprehensive and personalized prediction, incorporating key variables that influence margin status, and ultimately, patient survival.

The present study is limited by its retrospective design, which inherently carries limitations associated with data collection and potential biases. Additionally, the lack of external validation restricts the generalizability of our findings. Future research endeavors should prioritize prospective studies and external validations to overcome these inherent constraints and provide stronger evidence and reinforce the implications of the current study.

## 5. Conclusions

In conclusion, while *the essential is invisible to the eyes* [41], the application of a Bayesian model for preoperatively predicting microscopic margin status in PDAC resection after NAT offers a promising avenue. This model would provide a more personalized and precise approach for predicting surgical outcomes integrating individual patient characteristics and prior knowledge to enhance prognostic assessments.

## Figures and Tables

**Table 1:** Demographic and preoperative data.

Variable	n	All	R0, n 150	R1, n 55	*p*
Age, years (range)	205	64 (56–70)	62 (54–70)	66 (61–70)	0.018
Female, n (%)	205	100 (49)	70 (47)	30 (55)	0.3
BMI value, (range)	132	23 (21–27)	23 (21–27)	23 (21–27)	0.8
CCI > 4, n (%)	177	129 (73)	90 (68)	39 (89)	0.007
ASA score 3–4, n (%)	182	43 (24)	28 (21)	15 (31)	0.4
Jaundice, n (%)	196	94 (48)	62 (41)	32 (58)	0.014
Tumor site Head/Unc Process Body Tail	205	159 43 2	112 36 2	48 7 0	0.4
Neoadjiuvant chemotherapy, n (%) FOLFIRINOX Gem-Nab P Gem based	176	81 (46) 55 (31) 34 (19)	62 (47) 39 (30) 25 (19)	19 (42) 16 (36) 9 (20)	0.8
Neoadjuvant RT, n (%)	205	25	14 (27)	11 (20)	0.3
Preop vascular involvement (any) n (%)	190	108 (57)	70 (51)	38 (73)	0.006
Preoperative Ca19.9 U/mL (range)	205	22 (10–61)	23 (10–53)	21 (10–95)	0.3
Ca19.9 difference % (range)	154	81 (51–94)	86 (68–94)	63 (16–93)	0.020
Ca19.9 responders, n (%)	154	122 (79)	98 (64)	24 (16)	0.004
Preoperative normal Ca19.9, n (%)	181	101 (56)	80 (44)	21 (12)	0.14
Baseline tumor size, mm (range)	145	35 (29–40)	35 (30–40)	34 (28–40)	0.8
Preoperative tumor size, mm (range)	185	25 (20–30)	24 (18–29)	30 (23–35)	<0.001

Legend: BMI, body mass index; CCI, Charlson Comorbidity Index; ASA score, American Anesthesiologists Association score; RT, Radiotherapy.

**Table 2:** Perioperative and pathology details.

Variable	n	All	R0, n 150	R1, n 55	*p*
Venous resection, n (%)	205	49 (24)	28 (19)	21 (38)	0.004
I.O. blood loss, mL (range) I.O. transfusion, n (%)	172 170	350 (200–560) 27 (16)	300 (200–500) 12 (9)	450 (300–700) 15 (33)	0.006 0.001
Duration of surgery, min (range)	202	360 (295–420)	360 (293–415)	390 (325–485)	0.030
POPF grade B-C (n, %) PPH (n, %) PPH Grade 3 (n, %) Sepsis (n, %) DGE (n, %)	198 199 149 202 199	19 (9) 18 (9) 9 (6) 7 (3) 10 (5)	16 (10) 11 (7.5) 3 (3) 2 (1) 7 (5)	3 (6) 7 (13) 6 (13) 5 (9) 3 (6)	0.10 0.3 0.028 0.014 0.7
CD > 3a	200	28 (14)	13 (8,7)	15 (27)	0.001
Reintervention	202	18 (9)	9 (6)	9 (17)	0.027
Mortality	204	2 (1)	1 (0,7)	1 (1,9)	0.5
G, n (%) 0 1 2 3	204	15 (7) 3 (1) 162 (79) 24 (12)	15 (10) 3 (2) 115 (77) 16 (11)	0 (0) 0 (0) 47 (85) 8 (15)	0.033
T, n (%) 0 1 2 3	204	15 (7) 81 (40) 87 (43) 21 (10)	15 (10) 64 (43) 57 (38) 13 (9)	0 (0) 17 (31) 30 (55) 8 (15)	0.007
N, n (%) 0 1 2	204	92 (45) 69 (34) 43 (21)	78 (52) 44 (30) 27 (18)	14 (25) 25 (45) 16 (29)	0.003
Major path response (0–1)	200	52 (26)	48 (33)	4 (7)	0.001

Legend: POPF, postoperative pancreatic fistula; PPH, postoperative pancreatic hemorrhage; DGE, delayed gastric emptying; CD, Clavien-Dindo classification; G, grading; T, Tumor; N, nodes; M, metastases.

**Table 3:** Model summary with OR and 95% credible interval: the probability direction $p_{d}$ has also been reported.

Parameter	OR	95% CI	p_d_
Age	1.03	(1.01–1.06)	99.48%
ASA	1.70	(0.78–3.58)	90.83%
Arterial involvement	5.69	(3.83–8.50)	100.0%
Venous involvement	1.62	(0.78–3.45)	99.9%
Post -NAT size	1.08	(1.03–1.13)	89.6%
Primary tumor in the pancreatic body	2.79	(1.12–7.20)	98.4%
Preoperative normal Ca19.9	0.86	(0.42–1.75)	66.7%

Legend: ASA, American Anesthesiologists Association Score; NAT, neoadjuvant therapy.

## Data Availability

The data presented in this study are available on request from the corresponding author due to privacy reasons.

## Supplementary Materials

The following supporting information can be downloaded at: https://www.mdpi.com/article/10.3390/cancers16234106/s1, Figure S1: Area Under Curve (AUCs), Figure S2: Final Model Results.
