# Supplementary material for: Bayesan Model to Predict R Status After Neoadjuvant Therapy in Pancreatic Cancer
Source: Cancers (Basel). 2024 Dec 7;16(23):4106. doi: 10.3390/cancers16234106 (PMC11640340; doi:10.3390/cancers16234106)

Supplementary Material Figure S1

Area Under the Curve

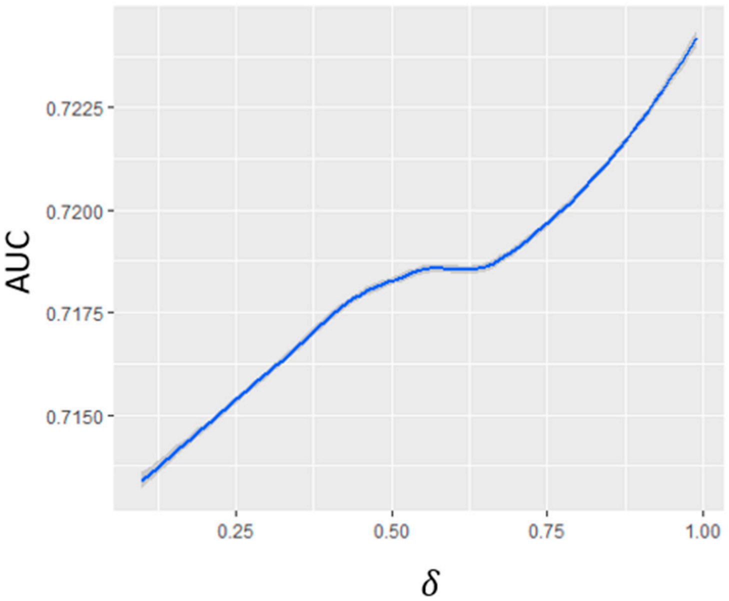

Area Under Curve (AUCs) estimated on the average Posterior Predictive probability according to discounting factor δ. The estimated AUCs have been interpolated with a Loess Function. The higher δ indicates a more discounted impact of the prior on the final inference.

Supplementary Material Figure S2

Final Model Results

Panel A
Model Summary with OR and 95% Credible Interval: the probability direction p_d has been also reported with the prior distributions.

Panel B
Log Odds with 90% and 95% Credible Intervals

Panel C
Posterior Probability of direction for predictors

Panel D
Trace plot

Panel A Model Summary (AUC 72%)

Parameter	OR	95% CI	p_d	Prior
Age	1.03	(1.01, 1.06)	99.48%	Normal (0.02 +- 0.02)
ASA_score	1.70	(0.78, 3.58)	90.83%	Normal (0.00 +- 7.14)
Arterial involvement	5.69	(3.83, 8.50)	100%	Normal (1.98 +- 0.23)
Post NAT size	1.08	(1.03, 1.13)	99.90%	Normal (0.12 +- 0.06)
Venous involvement	1.62	(0.78, 3.45)	89.65%	Normal (1.09 +- 0.72)
Tumor site (body)	2.79	(1.12, 7.20)	98.42%	Normal (1.70 +- 0.94)
Normal preop Ca19.9	0.86	(0.42, 1.75)	66.07%	Normal (0.90 +- 0.69)

Panel B Effect Plot

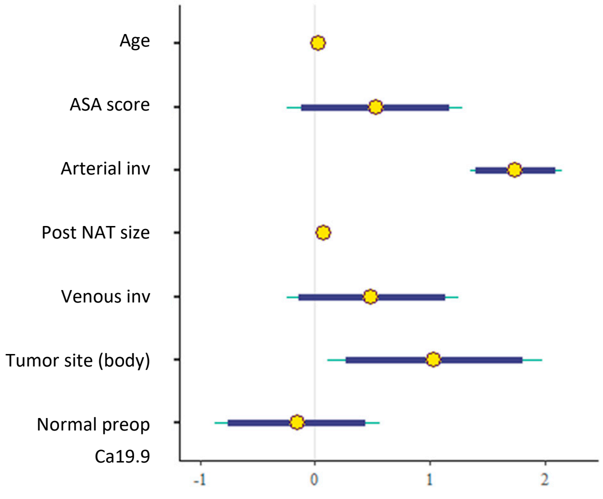

Panel C Probability of Direction

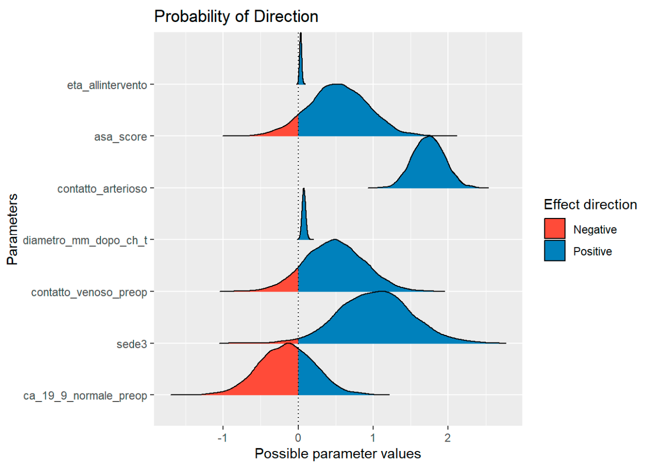

Panel D Trace Plot

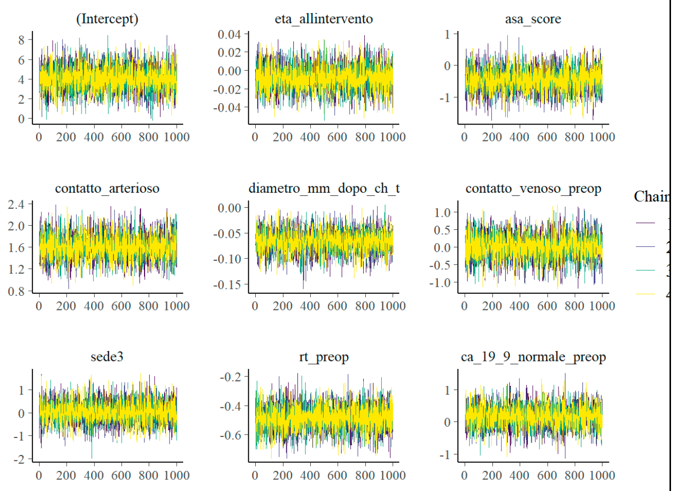

Supplement: Supplementary file 1 [file cancers-16-04106-s001.zip › cancers-3351934-supplementary.pdf]
